# Peptides from the Variable Region of Specific Antibodies Are Shared among Lung Cancer Patients

**DOI:** 10.1371/journal.pone.0096029

**Published:** 2014-05-01

**Authors:** Dominique de Costa, Ingrid Broodman, Wim Calame, Christoph Stingl, Lennard J. M. Dekker, René M. Vernhout, Harry J. de Koning, Henk C. Hoogsteden, Peter A. E. Sillevis. Smitt, Rob J. van Klaveren, Theo M. Luider, Martijn M. VanDuijn

**Affiliations:** 1 Department of Pulmonology, Erasmus Medical Center, Rotterdam, The Netherlands; 2 Department of Clinical Chemistry, Erasmus Medical Center, Rotterdam, The Netherlands; 3 StatistiCal BV, Wassenaar, The Netherlands; 4 Department of Neurology, Erasmus Medical Center, Rotterdam, The Netherlands; 5 Unit of Trials and Statistics, Erasmus Medical Center, Rotterdam, The Netherlands; 6 Unit of Public Health, Erasmus Medical Center, Rotterdam, The Netherlands; King’s College London, United Kingdom

## Abstract

Late diagnosis of lung cancer is still the main reason for high mortality rates in lung cancer. Lung cancer is a heterogeneous disease which induces an immune response to different tumor antigens. Several methods for searching autoantibodies have been described that are based on known purified antigen panels. The aim of our study is to find evidence that parts of the antigen-binding-domain of antibodies are shared among lung cancer patients. This was investigated by a novel approach based on sequencing antigen-binding-fragments (Fab) of immunoglobulins using proteomic techniques without the need of previously known antigen panels. From serum of 93 participants of the NELSON trial IgG was isolated and subsequently digested into Fab and Fc. Fab was purified from the digested mixture by SDS-PAGE. The Fab containing gel-bands were excised, tryptic digested and measured on a nano-LC-Orbitrap-Mass-spectrometry system. Multivariate analysis of the mass spectrometry data by linear canonical discriminant analysis combined with stepwise logistic regression resulted in a 12-antibody-peptide model which was able to distinguish lung cancer patients from controls in a high risk population with a sensitivity of 84% and specificity of 90%. With our Fab-purification combined Orbitrap-mass-spectrometry approach, we found peptides from the variable-parts of antibodies which are shared among lung cancer patients.

## Introduction

Lung cancer is currently the most common cancer with the highest mortality rate (28%) in the World due to diagnosis at an advanced stage.[1], [2] However, with the demonstration of a 20% lung cancer mortality reduction by the NLST trial (National Cancer Screening Trial) low dose CT screening for lung cancer is receiving increasing interest.[3] The NELSON trial (Dutch-Belgian lung cancer screening trial) showed that after three screening rounds 3.6% of all participants of this study had a false-positive screen result.[4] Although, still approximately 27% of the participants were subjected to invasive procedures that revealed benign lung diseases at baseline screening (first round NELSON trial).[5] A good biomarker (panel) will reduce this number of unnecessary invasive procedures. At the moment selection of high risk individuals for screening is done by age and smoking history. A biomarker or biomarker panel would be helpful in selecting high risk individuals for CT screening as this may detect lung cancer at an earlier stage than CT.

Antibodies can be interesting as markers for distinguishing lung cancer patients from lung cancer-free individuals. These antibodies are produced by the immune response that target specific tumor-associated antigens (TAAs) during cancer development, probably at an early stage.[6]–[12] Recently Liu et *al.* showed that the concentration of circulating IgG autoantibodies against ABCC3 transporter was significantly higher in female adenocarcinoma patients than in female controls [13].

Human antibodies consist of four chains, two identical heavy chains and two identical light chains. Each light chain has a variable (V_L_) and constant (C_L_) domain. The heavy chains have three different constant domains (C_H_1, C_H_2 and C_H_3) and a variable domain (V_H_). The first constant and variable parts form the antigen binding fragment (Fab). The remaining two constant parts of the heavy chain form the Fc region. Within the Fab six complementarity determining regions (CDR1, CDR2 and CDR3) are located between frameworks. These CDRs determine the antigen specificity and form a surface complementary to a shape that is part of the antigen. CDRs are hypervariable regions of the antibody.[14] Antibodies, or immunoglobulins, are highly complex molecules with large variation in their amino acid sequence. The possible diversity in immunoglobulins is estimated between 10^13^ and 10^50^ and therefore the finding of similar or even identical sequences in different individuals by chance is in theory, highly unlikely.[14], [15] However, studies of different research groups have recently demonstrated that despite this theoretical small chance to have identical antibodies among individuals, it is possible to identify similar or identical sequences.[16]–[19] A study performed by us showed that in PNS (paraneoplastic neurological syndrome) patients identical mutated primary amino acid sequences of complementarity determining regions (CDRs) exist. These CDRs are specific for known onconeural antigens, such as HuD and Yo in PNS patients, and most interestingly were shared between different PNS patients [20].

The aim of this study is to find evidence that specific antibody peptides are shared between lung cancer patients in contrast to lung cancer-free individuals. As lung cancer is a heterogeneous disease and with the variability of an antibody it might be a challenge to detect identical tumor-related antibodies in serum. We experimentally test the hypothesis that specific highly variable regions of an antibody including complementarity determining regions (CDRs) can be shared between lung cancer patients. Our experimental approach to verify this hypothesis is based on sequencing antibody peptides by mass spectrometry. Measurement of serum by a mass spectrometer might be too complex due to the high variability as mentioned above. Purifying IgG Fab from serum will reduce the complexity of the sample from a lung cancer patient and will give the possibility to focus on pure antibody fractions.

## Materials and Methods

### Ethics and Legal Approval

The NELSON trial was approved by the Dutch Health Council, the Minister of Health and by the Medical Ethical Committees of all participating centers (clinical trial number ISRCTN63545820). All participants for this study provided written informed consent for the use of their serum samples. The donor of the reference sample used throughout this study provided written consent for the use of his/her serum for scientific purposes according to the guidelines of the Blood Bank Sanquin, Rotterdam, the Netherlands.

### NELSON Trial

The NELSON (Dutch-Belgian Lung Cancer Screening trial) trial has started recruitment in 2003 by sending questionnaires to 548,489 males and females between 50–75 years of age. Participants had to be current or former smokers for at least 25 years, smoking at least 15 cigarettes per day or smoking at least 30 years, smoking at least 10 cigarettes per day. From the 548,489 males and females 15,822 participants were included in the trial. These participants were randomized to a screen or control arm. The screening arm received CT screening in years 1,2 and 4. The control arm received no screening (usual care). Participants with a positive test result were referred to a pulmonologist. If the diagnosis lung cancer was established the patient was treated and went off screening. Participants with an indeterminate test result underwent a follow-up scan three months later. If a negative test result was obtained the second-round CT scan was scheduled for 12 months later [5], [21].

### Study Population

For this study, we selected 44 lung cancer cases and 49 controls (Supplementary Figure S1) from the NELSON lung cancer screening trial.[5], [21] For the cases of the discovery set, NELSON 1, only early stage (I and II) squamous cell (n = 4) or adenocarcinomas (n = 21) were selected. They were carefully matched to the controls by age, gender, smoking status, duration and number of cigarettes smoked per day, chronic obstructive pulmonary disease (COPD) status, asbestos exposure and site of blood sampling (Supplementary Table S1). The selection criteria for the cases of the NELSON 2 (validation) set (n = 19) were similar, except that all non-small cell histology’s and disease stages were allowed (Supplementary Table S1) in order to challenge the results of the discovery phase. On purpose the clinical characteristics of the control patients are dissimilar with the NELSON 1 set in respect to smoking and COPD. Therefore, this NELSON 2 set is not matched with the NELSON 1 set. By using a validation sample set (NELSON 2) chosen in this way, the robustness of the method can be determined.

Serum samples were collected for both NELSON 1 and NELSON 2 obtained from baseline CT screening (first round).

### IgG Fab Purification and NanoLC Orbitrap MS Analyses

**Figure 1:**
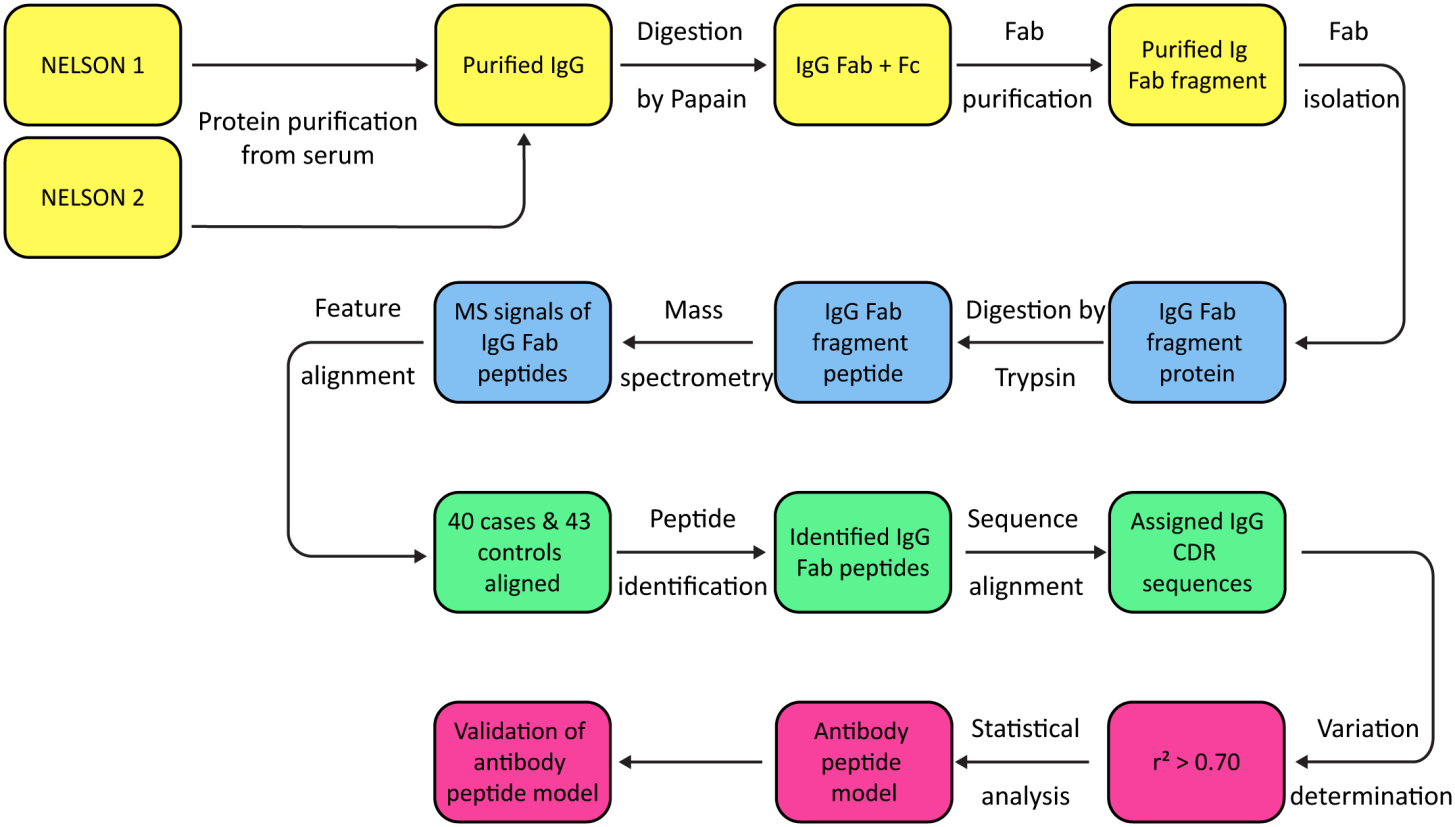
Flow-chart of the method and analysis used. In this flow-chart the different steps in Fab purification, Fab measurement and data analysis are illustrated. In yellow the Fab purification is shown, in blue the mass spectrometry measurement, in green the data analysis and in pink the statistical analysis.

Prior to all sample preparation procedures, all samples were blinded and the key for unblinding was put at the database coordinator of the NELSON trial. IgG Fab purification and nano-LC Orbitrap MS analyses were performed according to the method described before.[22] For a more extended description we refer to Supplementary Methods S1. In brief, IgG was isolated from serum and digested into Fab and Fc (Figure 1). The Fab part was isolated from the digested mixture by SDS-PAGE. The Fab containing gel bands were excised and tryptic digested. A blank piece of gel that was not loaded with protein was excised and treated like the excised Fab bands for background assessment.

LCMS measurements were performed on an Ultimate 3000 nano LC system (Thermo Fisher Scientific/Dionex, Amsterdam, the Netherlands) online coupled to a hybrid linear ion trap/Orbitrap MS (LTQ Orbitrap XL; Thermo Fisher Scientific, Bremen, Germany). 4 µL of the digested Fab was loaded onto the system. For further settings and solutions we refer to Supplementary Methods S1 and previous published work.[22] All samples were randomized before measurement and were measured in batches of 11 samples including a reference sample. A reference sample was used as a quality control for each measurement and analysis step. A blank sample was run at the start and end of the measurement to determine background and the existence of carry-over during chromatography.

### Data Analyses

Raw data files were loaded into the software Progenesis (Figure 1) (Version 3.1; Nonlineair Dynamics Ltd, New Castle, UK) and processes as described previously.[22] In addition, we performed a Progenesis analysis where instead of detecting features (peptide masses (m/z)) in all the samples at the same time by the software program, feature detection was performed individually per sample. Features picked thereby were matched to the Progenesis result table containing all samples with a mass tolerance of 5 ppm. This was of advantage, since often features occur with low intensities in one sample and are subsequently matched by Progenesis in all other samples. This result in errors related to background if one takes the respective mass spectra into account. With this relative small adjustment it ensures that a feature is detected more accurately throughout the samples. The data acquired by this approach was filtered using the same default settings.[22] A separate data matrix for every case and control was generated consisting of all features with corresponding raw abundance and retention time. To generate one large data matrix that includes all cases and controls from these separate data matrices, we searched masses from the separate data matrices per case or control in the complete data matrix generated from the standard Progenesis analyses. Every mass had to meet three criteria: 1) m/z (±5 ppm), 2) retention time (±1 min) and 3) identical charge. If a mass met these three criteria the raw abundance from the complete matrix (generated by a general procedure[22] recommended by the manufacturer) was used. If a mass did not meet these criteria a zero was generated for the raw abundance.

MS/MS spectra were extracted from raw data files and converted into Mascot compatible files using extract-msn (part of Xcalibur version 2.0.7, Thermo Fisher Scientific Inc.). Mascot (version 2.3.01; Matrix Science Inc., London, UK) was used to perform database searches against the human subset NCBInr database (version March 11^th^, 2009; Homo sapiens species restriction; 222,066 sequences) of the extracted MS/MS data (Figure 1). Database (NCBInr) dependent peptide identification and *de novo* sequencing results (software PEAKS; Version 5.2; Bioinformatics Solutions Inc., Waterloo, Canada) were also included in the Progenesis provided matrix. For settings used for the database search and *de novo* sequencing we refer to previous published work and methods S1.[22] For *de novo* sequences so far not known from a database, the Peaks software identifies a leucine for the isobaric amino acids leucine and isoleucine. Database dependent peptide identification results or *de novo* sequencing results were included in the matrix based on the highest peptide identity score (Data S1, Data S2 and Data S3). All peptide sequences from the cases and controls identified by Mascot or PEAKS were subsequently aligned to databases containing V, D, J or C-region germline sequences derived from IMGT database (IMGT, the international ImMunoGeneTics information system http://www.imgt.org) using the BLAST algorithm (Figure 1).[23] Peptides with sufficient match (bitscore ≥12.5 and alignment score ≥70%) to the V-region database were assigned to a position on the immunoglobulin molecule with varying CDR lengths (Data S1, Data S2 and Data S3).

Raw data files of the reference samples of each data set were separately loaded into the software Progenesis and followed the standard procedures as mentioned above. To determine the proportion of variation between the reference sample measurements performed on different time points, median r-squares were calculated for each sample. Each sample was compared to all the other reference samples measured in that dataset and a median r-square was calculated for each sample. The comparison was based on the raw abundance of each feature. This was performed separately for both independent datasets, NELSON 1 and NELSON 2 (Table S2a and S2b).

To determine the proportion of variation (Figure 1) between the samples (cases and controls) of the two separate datasets, the same calculations were performed as described above for each case and control sample. This analysis was performed separately for the two datasets (Table S2c and S2d). Based on the distribution of the median r-squares of each sample, we decided to set a cut-off at r-square >0.70. The cases and controls that obtained a median r-square below 0.70 were excluded from the dataset and further analyses. Calculations were conducted using Microsoft Excel 2007.

### Statistical Analysis

Two independent data sets have been used, NELSON 1 and NELSON 2. The initial step in the statistical analysis consisted of testing for normality using skewness and kurtosis distribution characteristics on the intensity of the raw abundance of the features [24].

Subsequently, univariate analysis was performed, applying either an unpaired t-test (parametric) or a Mann-Whitney U-test (non-parametric) to detect significant differences in raw abundance between cases and controls in the NELSON 1 set.[25] The significance limit was set at 0.05 (two-sided). All identified features that were found significantly different were used for the selection of features to distinguish lung cancer patients from controls.

Secondly, we used for multivariate analysis only the significantly identified features that had ≥2 triggered MS spectra. We applied a multivariate analysis on features fulfilling these criteria with a (logistic) stepwise regression model (y = a_1×1_ + a_2×2_ + a_3×3_….a_n_x_n_+ c) in combination with canonical linear discriminant analysis (Table S3a).[26], [27] This resulted in a combination of features with high sensitivity and specificity in the NELSON 1 dataset. This combination of features was then tested in the NELSON 2 dataset using the same methodology as described above.[26], [27] Note that for the NELSON 2 dataset it was necessary to optimize the coefficients in the model equation in order (Table S3b) to optimize the sensitivity and specificity in the NELSON 2 dataset.

To avoid a random-error effect in modeling, we verified the statistical background of the combination of features in a permutated dataset. The background evaluation consisted of the same workflow as used for the model building, except that at the beginning the assignment of cases and controls of NELSON 1 were permutated (Figure S2). This permutation was performed twelve times and the results obtained were tested for significance against the model outcome by z-test (one-sided; p<0.05). Since model building was based on the data as provided in NELSON 1 after which validation of this model was done using the data in NELSON 2, the same approach was taken after each individual permutation. Also here, note that for NELSON 2 dataset the coefficients in the model equation were optimized.

All analyses on model building, validation and background evaluation were done using STATA, version 12 (StataCorp, Texas, US). Throughout the study, using two-sided testing (except for one-sided testing for Z-values), p-values of 0.05 or lower were considered to be statistically significant. Statistical analyses of the data shown in Table S1 were generated by SPSS (IBM SPSS Statistics 20). The time to cancer was generated by calculating the interval between blood sampling and diagnosis for each case.

## Results

### Clinical Characteristics of the Study Population

There was no significant difference in the clinical characteristics between the cases and controls in the NELSON 1 set (Table S1). In the NELSON 2 set, current or former smoker and COPD status differed significantly between cases and controls (Table S1). In 72% and 84% of the cases of the NELSON 1 set, and NELSON 2 set, respectively, the time interval between blood sampling and lung cancer diagnosis was between 0–1.5 years. The median follow-up duration after blood sampling was for the control population 1925 days (range 1075–2086 days) and 1861 days (range 347–2135) in the NELSON 1 set and NELSON 2 set, respectively. None of the controls developed lung cancer during the follow-up period.

### Technical Variation

**Figure 2:**
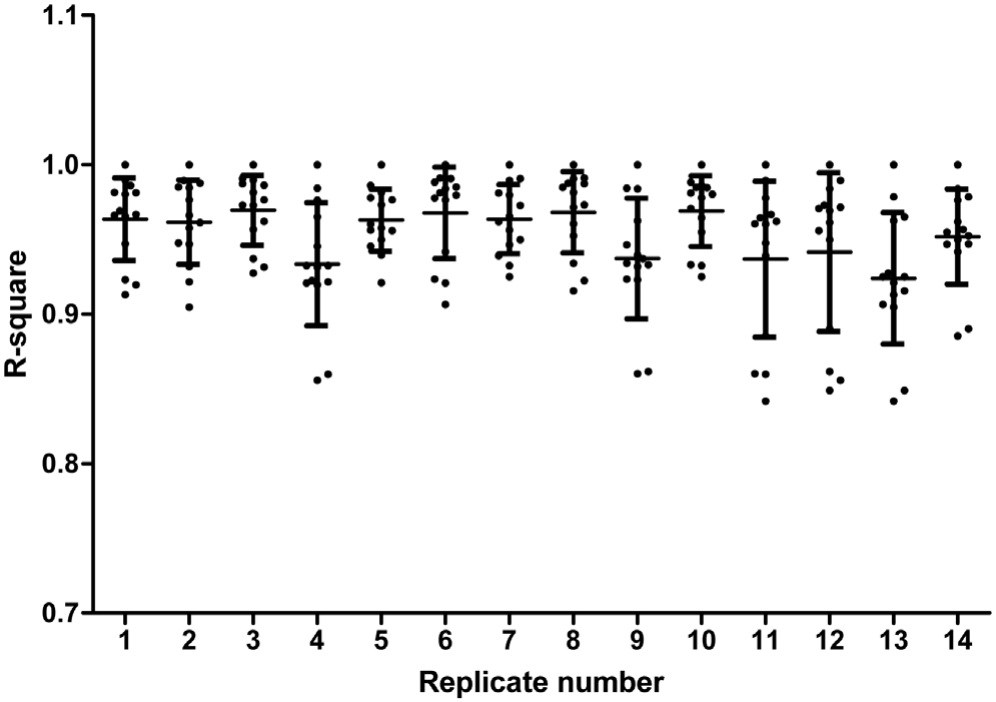
Technical reproducibility of replicate measurements of the reference sample. Reference sample measured at different time points during measurement of the NELSON 1 sample set. A replicate of the reference sample (x-axis) was compared to each other replicate sample based on the raw abundance of each feature. An r-square value was calculated. Each dot represents an r-square (y-axis) value for the comparison of that specific replicate with another replicate. For each replicate the average r-square and standard deviation (SD) is shown.

During the mass spectrometry measurements of the biological samples we measured a reference sample at different time points. R-square values were calculated from the abundances of identified proteins in each reference measurement to show technical reproducibility. The lowest r-square value observed in the different measurements ranged between 0.84 and 0.93 (Figure 2).

**Figure 3:**
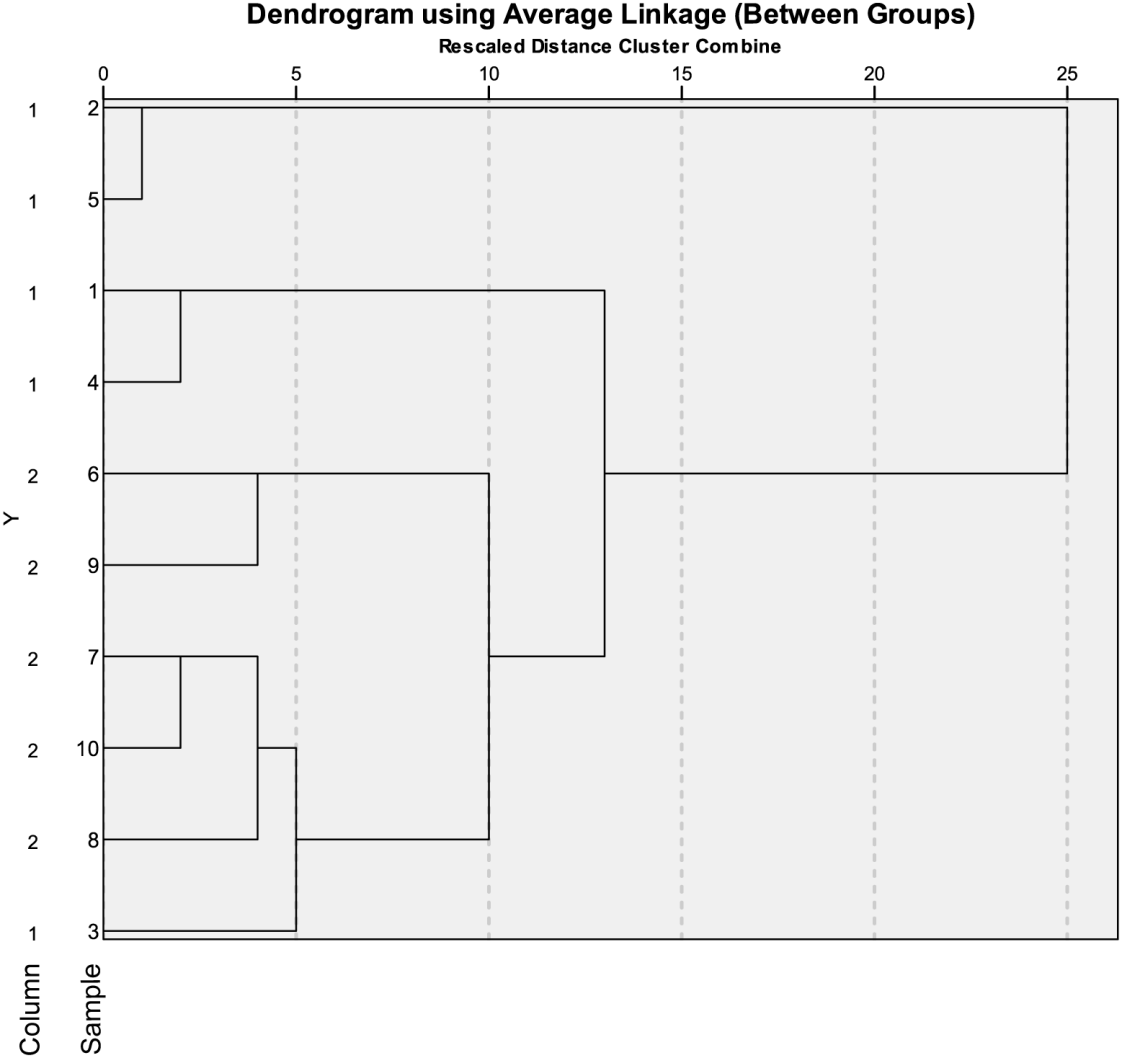
Technical reproducibility of five biological samples measured on two different columns at different time points. This dendrogram shows the correlation between five different biological samples measured on two different columns from same batch, column 1 and column 2 (y-axis). On the y-axis the five different samples are shown. Sample 1–5 are measured on column 1 and 6–10 are measured on column 2. Sample 1 and 6 are from the same individual. This also applies for sample 2 and 7, 3 and 8, 4 and 9 and 5 and 10. On the x-axis the Euclidian distance between each sample is shown. A strong correlation per column is found.

We performed the same r-square calculation for 5 random biological samples taken from the NELSON 1 set that were measured on two different LC-columns (same batch) at different time points. The technical reproducibility within each column resulted in lowest r-square values ranging from 0.75–0.93, but the technical reproducibility of the five biological samples measured on two independent similar columns was lower. For the two independent similar columns a median r-square of 0.52 was observed. In Figure 3 the correlation between each sample and between columns are shown.

**Figure 4:**
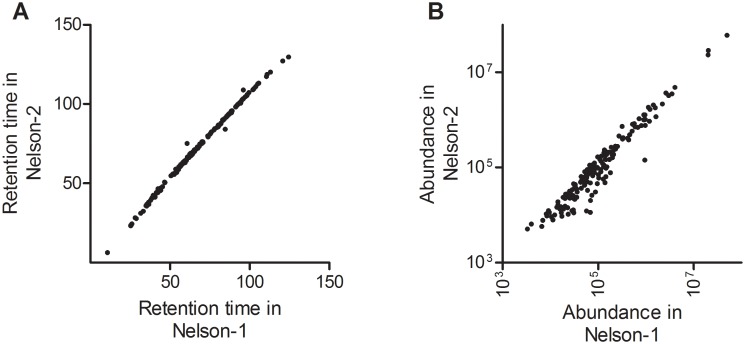
LC-MS performance for high abundant peptides in Nelson 1 and Nelson 2. For Reference samples that were measured during both NELSON 1 and NELSON 2, we compared peptides that were identified with high confidence by a Mascot search with a score of more than 60 in both sets. For this subset of peptides, we compared the retention times observed in Nelson 1 and Nelson 2 (A) and also their abundance (B). For these parameters we observed r-square values of 0.996 and 0.995, respectively.

In Figure 4A the retention times are shown for peptides identified with high confidence (Mascot score >60) in the Reference samples measured concurrently with both NELSON 1 and NELSON 2. This Figure shows that column performance was comparable between the two different LC columns for these abundant peptides (r-square 0.996). In addition, the abundances observed for these peptide also correlated well (Figure 4B; r-square 0.995). This suggests that both chromatography and mass spectrometry performed nominally, at least for peptides identified with high confidence at relatively high abundance. Thus, the technical variation we see primarily stems from peptides at lower abundances, closer to the detection limits (Figure S3).

An estimation of the biological variation was performed and resulted in a median r-square of 0.43. This result was much lower than the lowest r-square (0.84) observed for the technical variation. Therefore, the biological variation is higher compared to the technical variation.

These results show that technical variation should be taken into account and adjustment is needed for comparison of independently measured sample sets since the NELSON 1 and NELSON 2 dataset were measured on two different columns at different time points. To overcome this technical variation, we applied a number of filters on the data before we could start a data analysis as described in the Material & Methods section.

With this data we performed separate univariate analysis on all peptides found in cases and controls from the separate NELSON 1 and NELSON 2 data set. We were able to observe 49 peptides that were significantly different between cases and controls in the NELSON 1 dataset. However, these peptides, with one exception, did not show this difference in the NELSON 2 dataset. There was no trend observed (r-square 0.004) in p-values for the two datasets. Therefore, testing univariately in this manner was either not the right analysis strategy or the process generated randomly selected features (chance). Therefore, the significant peptides from NELSON 1 were analyzed as a next step in a multivariate way.

### Antibody Peptide Model

**Figure 5:**
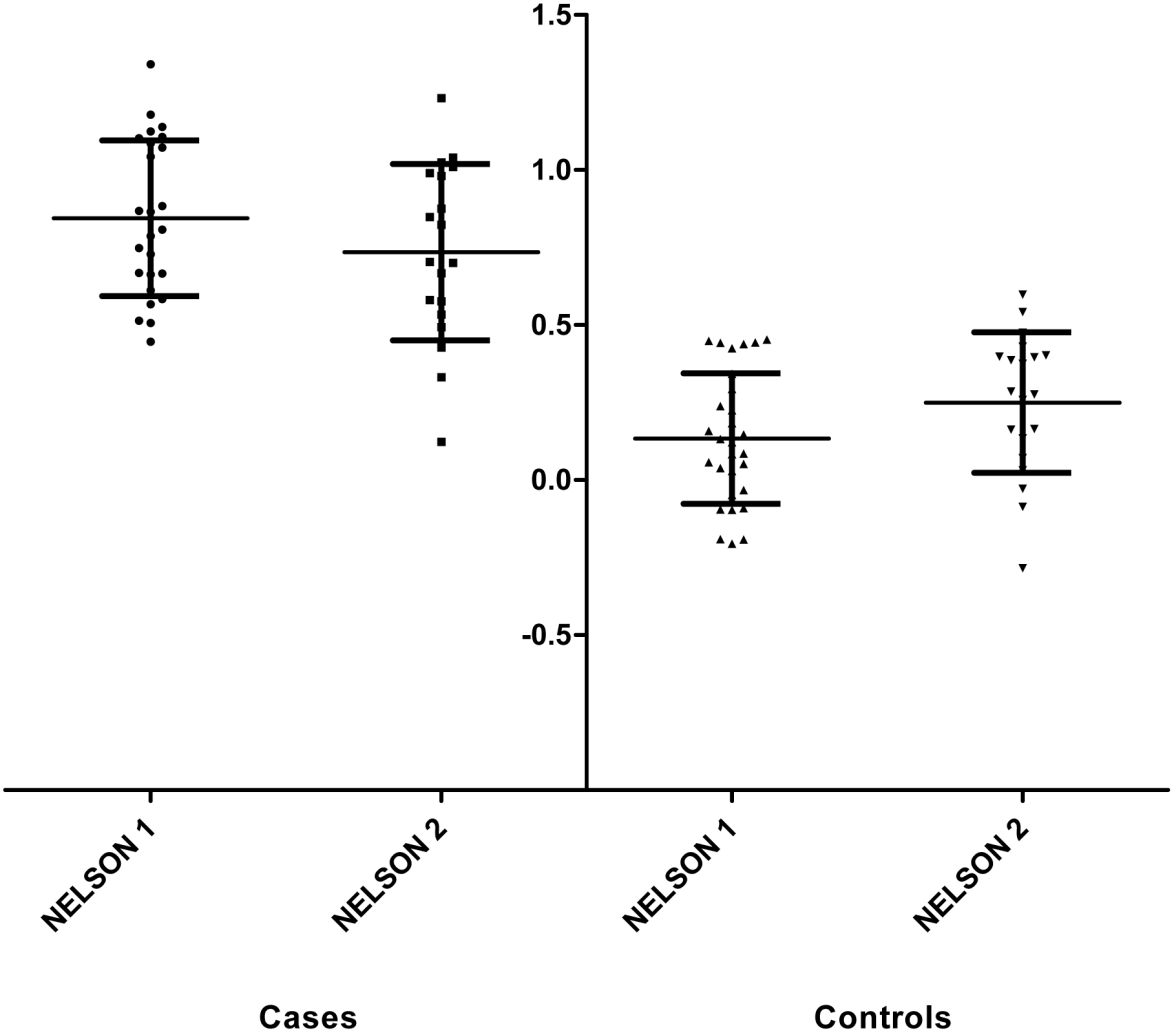
Distribution of the antibody peptide model outcome of the NELSON 1 and NELSON 2 sets. The raw abundances are filled-in in the model equation (y = a_1×1_ + a_2×2_ + a_3×3_….a_n_x_n_+ c) of the relevant sample set. On the y-axis (in arbitrary units) the figures generated by the equation are shown.

An optimal combination of 12 peptides was identified by the multivariate statistics used on the NELSON 1 set (discovery set). This combination of peptides could distinguish lung cancer patients from controls with sensitivity and specificity of 96% and 100%, respectively. This antibody peptide model was able to detect lung cancer 373 days on average (range 39–1193 days) before the diagnosis was determined. In Figure 5 we show that the combination of the 12 peptides was able to distinguish cases from controls. The 12 peptides corresponded to 1 sequence overlapping with the CDR2 region, 1 sequence overlapping CDR3 region, 7 sequences overlapping the Framework 1 region and 3 sequences overlapping with the Framework 3 region according to the IMGT database (Table 1).

**Table 1:** Information of the 12 peptides of the antibody peptide model.

Peptide	CDR/Framework	Sequence	m/z	Charge	Protein description	BLAST bit score	IMGT % Identified	p-value
1	Fr1	GITLSVRP	421.758	2	gi|553734: T-cell receptor	16.8	83.3	0.045
2	Fr3	LMAWLDLK	503.274	2	De Novo Peptide	15.9	75.0	0.009
3	CDR2	IYWDDDKR	555.763	2	gi|39938054: Immunoglobulinheavy chain variable region	32.9	100.0	0.039
4	Fr3	SYPLTFGGGTK	564.288	2	gi|4378188: Immunoglobulinkappa variable region	26.5	100.0	0.014
5	CDR3	LLLYTGGDQR	568.301	2	De Novo Peptide	18.0	75.0	0.030
6	Fr1	EVLLVESGGGLVKPGGSLR	623.025	3	gi|2072264: Immunoglobulinheavy chain	53.2	94.7	0.017
7	Fr3	NTVFLEMNSLR	670.336	2	gi|112699425: Immunoglobulinheavy chain variable region	32.9	81.8	0.040
8	Fr1	HVQLQESGPGLVK	696.386	2	De Novo Peptide	39.7	92.3	0.031
9	Fr1	SYSCQVTHEGSTVEK	827.872	2	gi|16554039: Immunoglobulinheavy chain	50.7	75.0	0.036
10	Fr1	SELTQDPAVSVALGQTVR	936.000	2	gi|87901: Immunoglobulinlambda variable region	57.1	100.0	0.030
11	Fr1	VSSVRCTSGGGLVQPGGSLR	959.501	2	gi|112702369: Immunoglobulinheavy chain variable region	40.1	100.0	0.042
12	Fr1	REMTKPPSVSVSETSHR	964.487	2	De Novo Peptide	29.1	81.8	0.013

Footnote: Fr: Framework; CDR: Complementarity Determining Region.

We performed an external validation in the NELSON 2 (validation) set. When we applied the same 12 peptide model to this set, cases and controls could no longer be distinguished. However, with the same peptides but after re-optimization of the model coefficients, we observed a sensitivity and specificity of 84% and 90%, respectively. As the coefficients of the equation are adjusted we had to check for the chance of overfitting of the data. Therefore, a background evaluation was performed which will be described later. Within the NELSON 2 validation set the combination of peptides was able to detect lung cancer 281 days on average (range 54–777 days) before the diagnosis of lung cancer.

We compared the raw abundance of the 12 peptides between the two NELSON datasets. We observed that the average raw abundance of five peptides was higher in the cases compared to the average abundance of the controls from the NELSON 1 dataset. These data were consistent with the findings from the NELSON 2 dataset (Table S4). The other seven peptides had a higher average raw abundance in the controls of the NELSON 1 dataset compared to the abundance in the cases of this dataset. For only one of these seven peptides, this difference could be confirmed in the NELSON 2 dataset (Table S4).

### Background Evaluation of Antibody Peptide Model

**Figure 6:**
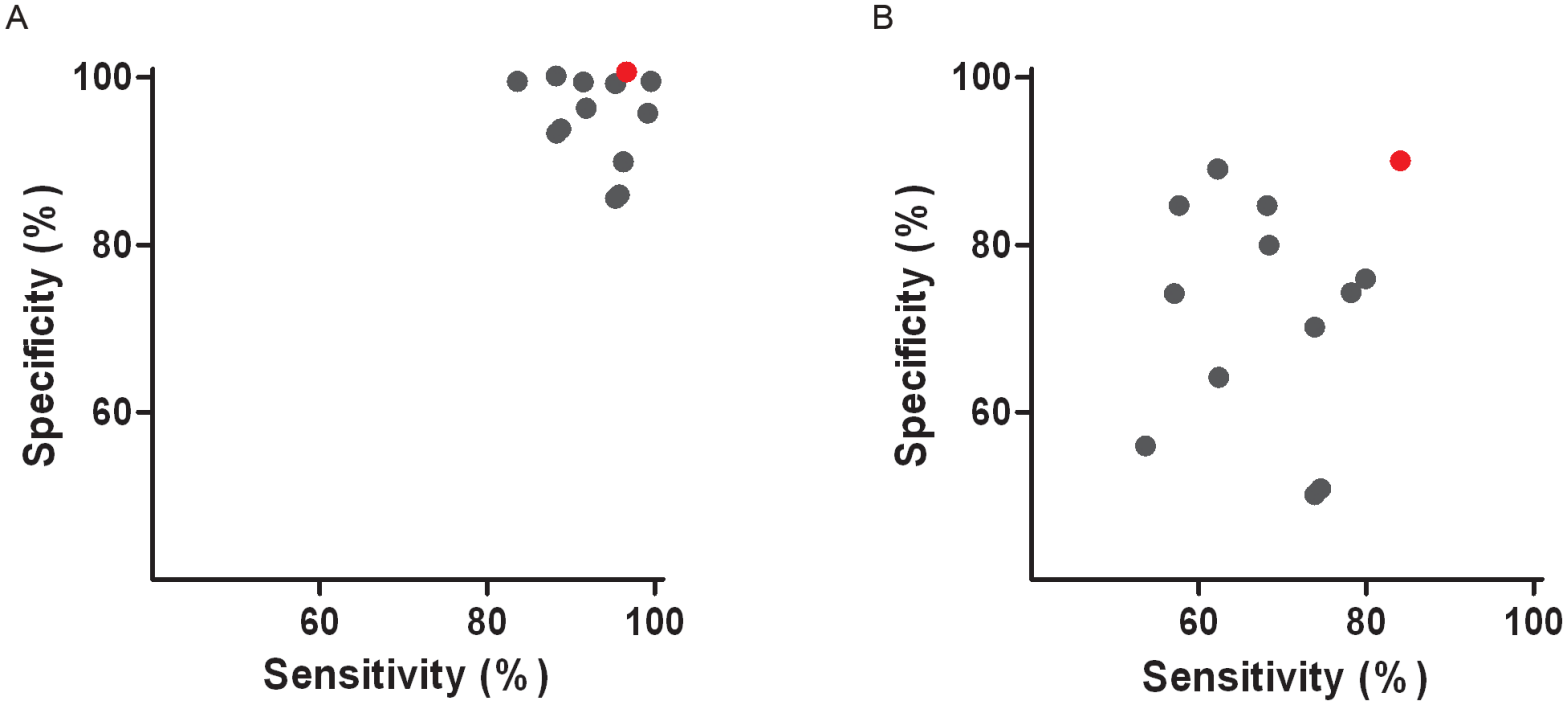
Background determination in NELSON 1 and NELSON 2 datasets. Twelve times a permutation (Background) was performed on the NELSON 1 and NELSON 2 dataset. The sensitivity and specificity of the antibody peptide model are shown in red. Background assessment: **A**) Twelve permutation runs are shown with the corresponding sensitivity and specificity of the NELSON 1 dataset (blue). The same 12 peptides found in the background evaluation of NELSON 1 were tested in NELSON 2. **B**) The 12 runs are shown with the corresponding sensitivity and specificity of NELSON 2 dataset (blue). Note, as some results of the background analysis occurred more than once, a random number between -1 and 1 were added to each sensitivity and specificity number to make sure each analysis (blue dot) can be seen in the figure.

In addition to the finding of the optimal combination of peptides which significantly distinguished cases from controls, a background analysis was performed. As the coefficients of the equation of the model were adjusted for each dataset we verified the results for a contribution of random selection of the data and thereby the chance of finding a comparable model by chance. The same workflow was applied for the model building except that at the beginning of the workflow the cases and controls of NELSON 1 were permutated at random (Figure S2). Discovery was performed in the 12 times permutated NELSON 1 datasets, each time with 12 different peptides showing the lowest p-value (p<0.05) in the NELSON 1 set for that particular permutation. Validation of these models was performed in NELSON 2. The performance of the multivariate model of the permutated discovery sets (NELSON 1) is shown in Figure 6A (blue dots) where the sensitivity is plotted against the specificity. The corresponding power in the validation sets (NELSON 2) is shown in Figure 6B (blue dots). Thus, each point in Figure 6A (blue dot) corresponds with a point (blue dot) in Figure 6B. Also, the performance found for the actual datasets in which the antibody peptide model was found is plotted (red dot). It can be observed that the multivariate fitting from the permutated datasets produces reasonable models even for permutated data in the discovery set.

However, especially in the validation datasets, the real data (antibody peptide model) performed significantly better (p<0.05) than the permutated datasets, suggesting that the immunoglobulin peptides harbor information related to the disease state of the patient. Thus, the results we obtained do not stem from an artifact in the data processing.

### CT Screening Result in NELSON 1 and NELSON 2 Dataset

**Figure 7:**
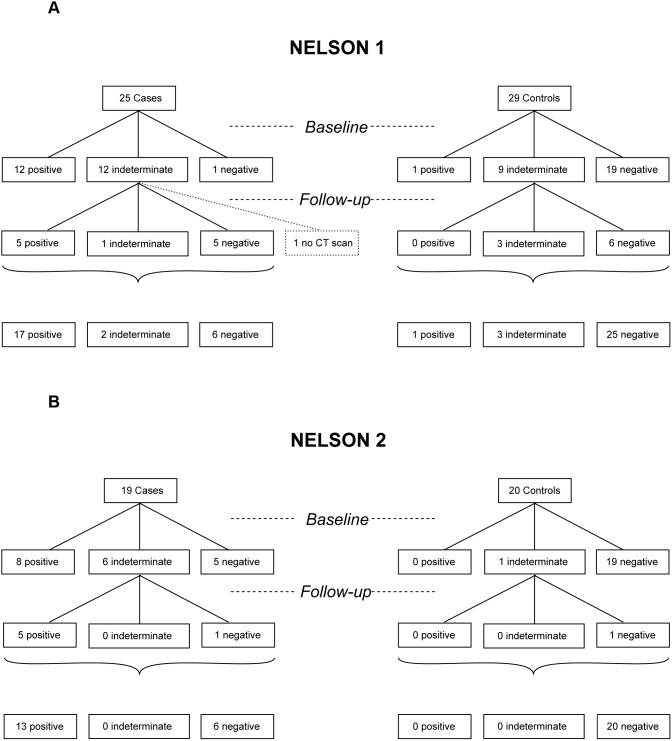
CT scan results of the NELSON 1 and NELSON 2 sample set. CT scan results of the **A**) NELSON 1 and **B**) NELSON 2 sample sets are shown at time of blood sampling (Baseline). Also, CT results are shown of the follow-up CT scan after approximately three months (Follow-up). For one case from the NELSON 1 set no Follow-up CT scan result was available. The last row represents the numbers of positive, indeterminate and negative CT scan results of baseline including follow-up results.

In Figure 7A and 7B the screening results of the baseline CT scans are shown for the NELSON 1 and NELSON 2 set, respectively. According to the screening protocol of the NELSON trial, a repeat CT scan was performed following an indeterminate screening result, approximately 3 months later.

We observed that 68% of the cases had a positive screening result in both the NELSON 1 and NELSON 2 set during the first 3 months of the screening program, the other lung cancers were diagnosed following another repeat CT scan after 3 months or during the second screening round. After on average 367 days (range 39–1193 days) for NELSON 1 and 269 days (range 54–777 days) for NELSON 2, the screening result was positive, i.e. suspect for lung cancer and resulting in clinical work-up by the pulmonologist and eventually finally diagnosis of lung cancer.

## Discussion

By mass spectrometry we found evidence that a proportion of peptides of the variable part of antibodies differ between lung cancer patients and controls. A combination of 12 different peptides was able to distinguish lung cancer patients from controls in a high risk population. A sensitivity of 96% and a specificity of 100% were observed in the discovery set. An external validation in an independent case–control set was performed and generated a sensitivity of 84% and a specificity of 90%. The background evaluation showed that the 12 antibody peptide model performed significantly better than a model generated based on permutated data.

Recently, Arentz et al. published that uniquely mutated V regions peptides could be used as a proxy for the detection of anti-Ro52 autoantibodies in sera from primary Sjögren’s syndrome patients by mass spectrometry.[28] Why these and other studies were able to identify similar or identical sequences could be explained by repertoire bias and the convergent evolution of antibodies during somatic mutation and selection.[19], [20] This selection favors specific alleles and sequences of antibodies with the optimal affinity towards the specific antigens during immune response [18], [29], [30].

We were able to identify peptide sequences which were distributed differently between lung cancer patients and controls. The antibody peptide model consisted not only of peptide sequences positioned at the CDR regions of an immunoglobulin but also at the framework region surrounding the CDRs. It may appear surprising that most of the peptides that are represented in the antibody peptide model derive from framework regions of the immunoglobulin, rather than from the hypervariable CDRs. This may be explained by their abundance in the immunoglobulin pool. Peptides carrying only few mutations relative to the germline are more likely to occur in several antibody clones, and thus have a higher abundance. This favors their detection by the mass spectrometer, especially in samples of high complexity. While technological advances may enable the reliable quantitation of also lower abundant peptides, it may even be that hypermutated CDRs are not as likely to be common among patients sharing an immune response. But moderately mutated peptides strike the best balance between specificity, abundance and sharing for the purposes of a diagnostic marker. The large heterogeneity of lung cancer could also contribute to the presence of fewer CDR peptides shared between lung cancer patients.

We observed that the average raw abundance of 6 from the 12 peptides was distributed differently in the cases versus controls between the two datasets. The average raw abundance of these six peptides was higher in the controls in the NELSON 1 set but in the NELSON 2 set the average raw abundance was higher in the cases. This may be due to the increased technical variation we observed for lower abundance peptides between the sets that were measured some time apart on different LC columns. While the system operated nominally for abundant peptides, possibly the performance close to the detection limit cannot be held constant over time, affecting reliable detection and quantification of such peptides.

For our validation set, NELSON 2, we used all disease stages in contrast to NELSON 1. In NELSON 1 we only used early stage I and II. Using different stages of lung cancer could also contribute to the average raw abundance discrepancies between NELSON 1 and NELSON 2. It could be that tumor-specific antibodies are more abundant in sera from early stage lung cancer patients compared to late stage lung cancer patients. We repeated our data analysis for cohorts that were a mixture of Nelson 1 and -2 data. While this reduced the clinical differences between the Discovery and Validation sets, advantages from this improvement were outweighed by the technical differences between the samples. While similar trends were observed, they were not as strong as those shown in Figure 6 (Figure S4).

We also have to cope with the high variability of immunoglobulins, which make the samples probably too complex for the mass spectrometer. A solution to this problem could be reduction of the complexity of the sample before it is measured on the mass spectrometer. This reduction could be established by fractionation into smaller protein fragments such as Fab-κ and Fab-λ, or by producing immunoglobulin fragments containing just the variable domains of the IgG molecule.

It was our aim to offset biological variation by including a relatively large number of patients in this study, but unfortunately large sample numbers translate to extended measurement times of up to 8 weeks for a dataset. These measurement times introduce technical variation that counteracts the advantage gained from the number of included patients.

We were not able to distinguish lung cancer cases from controls univariately by one peptide. Instead we needed a panel of different peptides to discriminate significantly between cases and controls. Lung cancer is a very heterogeneous disease which results in high variability between patients and cancer types. This might induce various immune responses to different tumor antigens.[6]–[12] Therefore, finding only one antibody that is shared between all lung cancer patients is highly unlikely. Brichory et al. for instance showed for PGP 9.5, annexin I and II a sensitivity of only 14%, 30% and 33%, respectively.[31], [32] Chapman et al. tested a panel of seven TAAs and found a sensitivity of 41% and a specificity of 93%. Validation of this panel in an independent sample set showed a sensitivity and specificity of 47% and 90%, respectively.[33] Koziol et al. were able to distinguish lung cancer patients from normal individuals with a panel of seven TAAs. A sensitivity of 80% and a specificity of 90% were observed, but no validation was performed.[34] Moreover, Khattar et al. and Zhong et al. were able to identify validated autoantibody peptide panels for lung cancer screening with sensitivity and specificity ranging from 84%–91% and 73%–91%, respectively.[35], [36] It is therefore not surprising that no single peptide could be found in the current data set that distinguishes cases from controls.

Using a multivariate model, we were able to distinguish lung cancer patients from controls. However, due to the experimental and biological variation, it was necessary that we recalibrated our model for each group of patients. This limits the current applicability of the method in the clinical practice, at least until significant technical advances enable a more robust quantification and identification of peptides in such complex samples. Still, we conclude from our data that differences exist between the immunoglobulin-derived peptides from early lung cancer patients and controls. This is corroborated by data from earlier studies in our own group as well as in other research groups that showed conservation and sharing of rearranged immunoglobulin sequences in immunoglobulins against a particular antigen [19], [20], [28].

So far, only age and smoking history have been used as selection criteria for enrolment in screening trials, but it is well known that even though over 80% of all lung cancer cases are directly related to smoking, only 11% of female smokers and 17% of male smokers will be diagnosed with lung cancer during their lifetimes.[37], [38] Therefore, additional diagnostic tests might select high risk individuals more precise when combined with the selection criteria age and smoking history in screening trials. The cases and controls we used for this study were selected based on their diagnosis of lung cancer within three years (range 39–1193 days) after the baseline CT scan. Therefore, calculation of sensitivity and specificity of CT screening in our subset of cases and controls from the NELSON trial are not applicable in this retrospective study. However, in this study we have demonstrated that 68% of the cases were detectable for lung cancer by CT screening. At the same time point the CT scan was performed, the antibody peptide model was able to detect lung cancer in 96% and 84% of the cases in the NELSON 1 and NELSON 2 set, respectively. Eventually after approximately 1 year the screening result of all cases were positive by CT screening.

In the high risk population of the NELSON trial still approximately 27% of the participants are subjected to invasive and expensive follow-up studies that revealed in benign disease at baseline CT screening.[5] The performance of CT improves after follow-up scans, but only after an amount of time has passed, on average a year for the sets in this study. Thus, there is need for additional diagnostic capabilities that can improve the performance of the current testing at baseline. For example, the group of Massion recently published their results on a combination of a serum proteomic biomarker panel with clinical and CT data.[39] In the current study, we were able to detect lung cancer with an antibody peptide model in the NELSON 1 and NELSON 2 set with sensitivities of 96% and 84% and specificities of 100% and 90%, respectively at an early stage. This indicates that specific antibodies are present at an early disease stage and that such a panel of antibodies is able to detect lung cancer at an earlier stage than CT. Auto-antibody profiling has the potential to be a tool for early detection when incorporated into a comprehensive screening strategy if technical challenges described in this study can be overcome.

In conclusion, a panel of antibody peptides is identified that discriminates samples of lung cancer patients from controls. This is a first indication that peptides generated from the variable part of antibodies are shared between lung cancer patients and can be used to discriminate lung cancer patients and control groups. More quantitative work is still needed to assess the use of these peptides in clinical settings.

## Supporting Information

Figure S1 **Study Flow-chart.** A flow-chart diagram of the samples used in this study. NSCLC: Non-small cell lung carcinoma.
(TIF)

Figure S2 **Statistical analysis flow-chart.** Before background analysis is performed, cases and controls of the NELSON 1 dataset are shuffled randomly.
(TIF)

Figure S3 **Variation at different abundances.** The abundances of all peptides in the reference sample compared in data from the Nelson-1 and Nelson-2 datasets. Superimposed, the subset of peptides that was identified with high confidence, as plotted in Figure 4B, has been superimposed in red.
(TIF)

Figure S4 **The performance of the prediction model was tested in Training and Testing sets, for both real data, and data in which the assignment of cases and controls had been randomized.** This approach is the same as in Figure 6, except that each set was composed of samples drawn from a combination of both the Nelson-1 and Nelson-2 sets. We assessed three such combinations, and four permutations.
(TIF)

Table S1 **Clinical characteristics of the NELSON 1 and NELSON 2 sample sets.**
(XLS)

Table S2 **A–D. R-square of all reference and clinical samples of NELSON 1 and NELSON 2.**
(XLS)

Table S3 **A–B. Regression and canonical discriminant analyses of NELSON 1 and NELSON 2 datasets.**
(XLS)

Table S4 **Raw abundance of the 12 antibody peptides in the NELSON 1 and NELSON 2 dataset.**
(XLS)

Data S1 **Data matrix consisting of peptide features detected by the Progenesis analysis software, the abundances of these features in the serum samples, annotation with peptide sequences and alignment results with an immunoglobulin sequence database.** The data was divided into three segments to improve tractability, this is segment one.
(ZIP)

Data S2 **Data matrix consisting of peptide features detected by the Progenesis analysis software, the abundances of these features in the serum samples, annotation with peptide sequences and alignment results with an immunoglobulin sequence database.** The data was divided into three segments to improve tractability, this is segment two.
(ZIP)

Data S3 **Data matrix consisting of peptide features detected by the Progenesis analysis software, the abundances of these features in the serum samples, annotation with peptide sequences and alignment results with an immunoglobulin sequence database.** The data was divided into three segments to improve tractability, this is segment three.
(ZIP)

Methods S1 Additional detail on sample collection, the study population, IgG Fab Purification, NanoLC Orbitrap MS analyses and *de novo* sequencing.
(DOCX)
