# Supplementary figures and images for: Peptides from the Variable Region of Specific Antibodies Are Shared among Lung Cancer Patients
Source: PLoS One. 2014 May 1;9(5):e96029. doi: 10.1371/journal.pone.0096029 (PMC4006902; doi:10.1371/journal.pone.0096029)

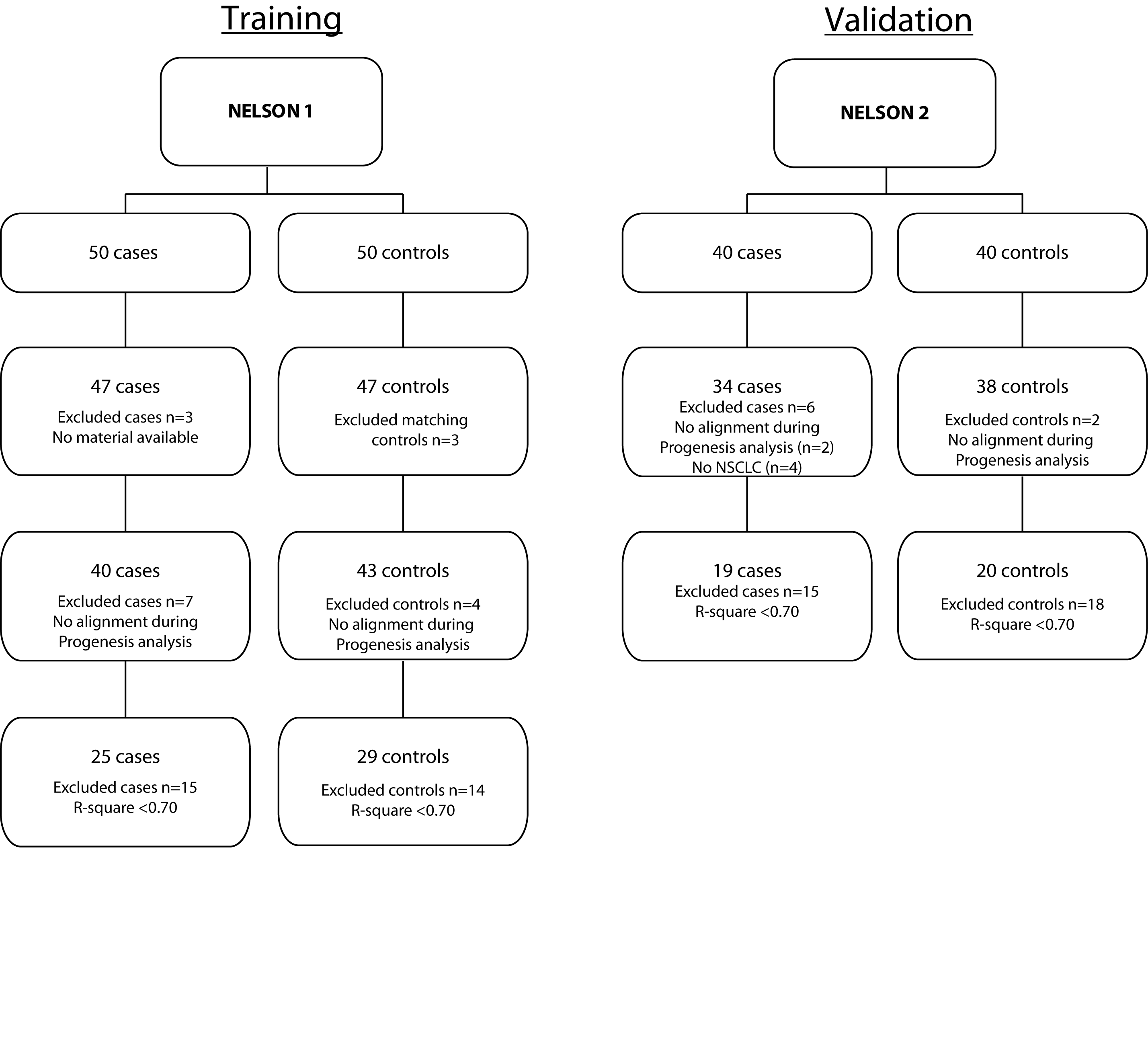

Supplement: Figure S1 — Study Flow-chart. A flow-chart diagram of the samples used in this study. NSCLC: Non-small cell lung carcinoma. (TIF) [file pone.0096029.s001.tif]

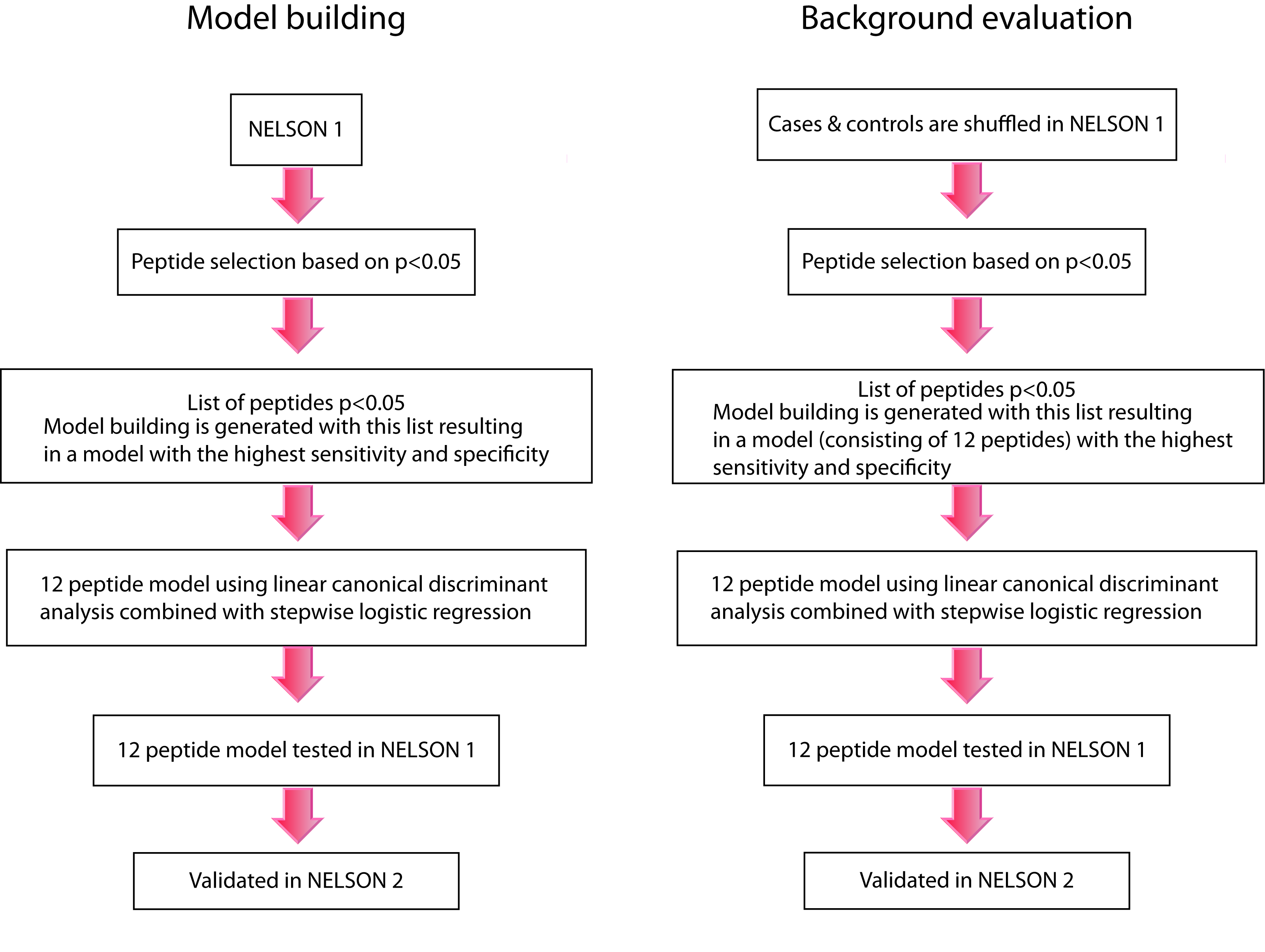

Supplement: Figure S2 — Statistical analysis flow-chart. Before background analysis is performed, cases and controls of the NELSON 1 dataset are shuffled randomly. (TIF) [file pone.0096029.s002.tif]

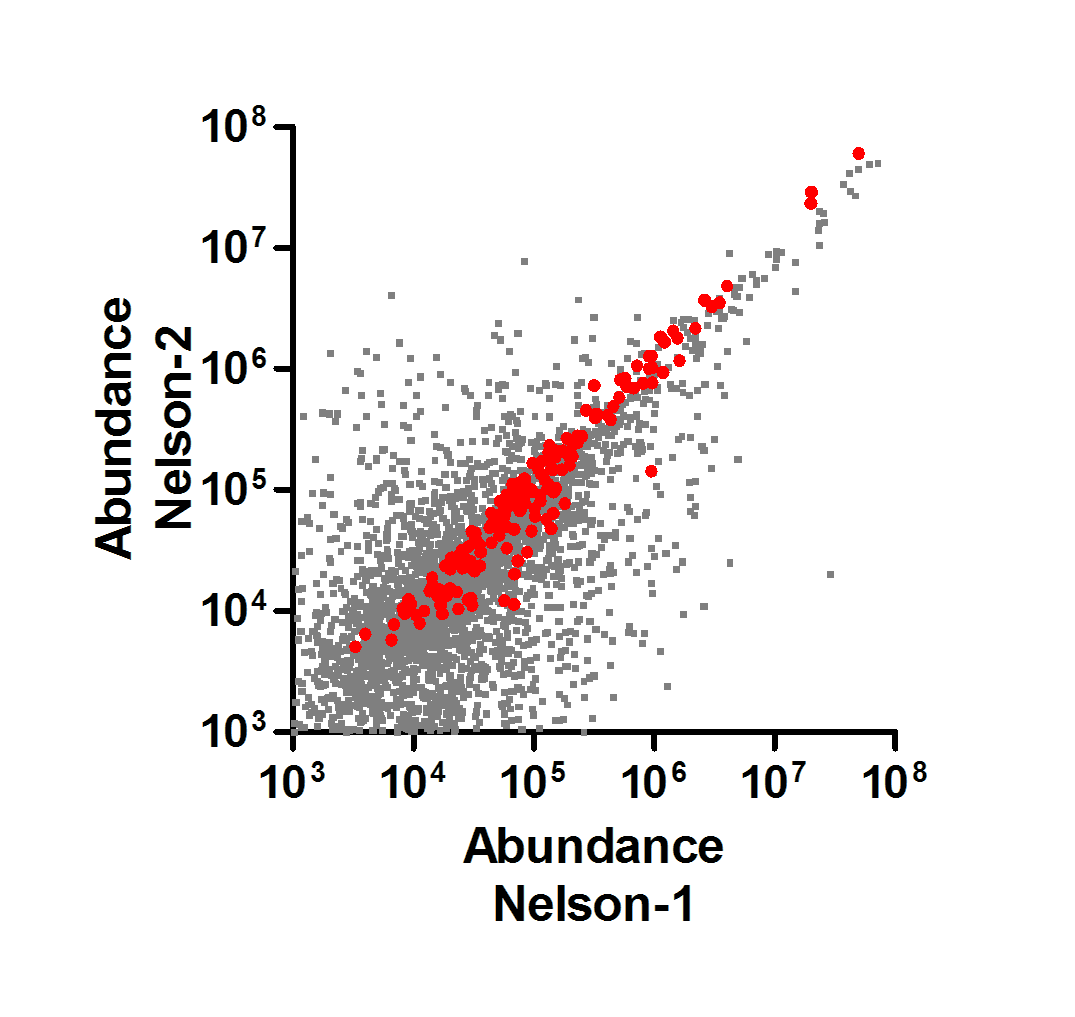

Supplement: Figure S3 — Variation at different abundances. The abundances of all peptides in the reference sample compared in data from the Nelson-1 and Nelson-2 datasets. Superimposed, the subset of peptides that was identified with high confidence, as plotted in Figure 4B, has been superimposed in red. (TIF) [file pone.0096029.s003.tif]

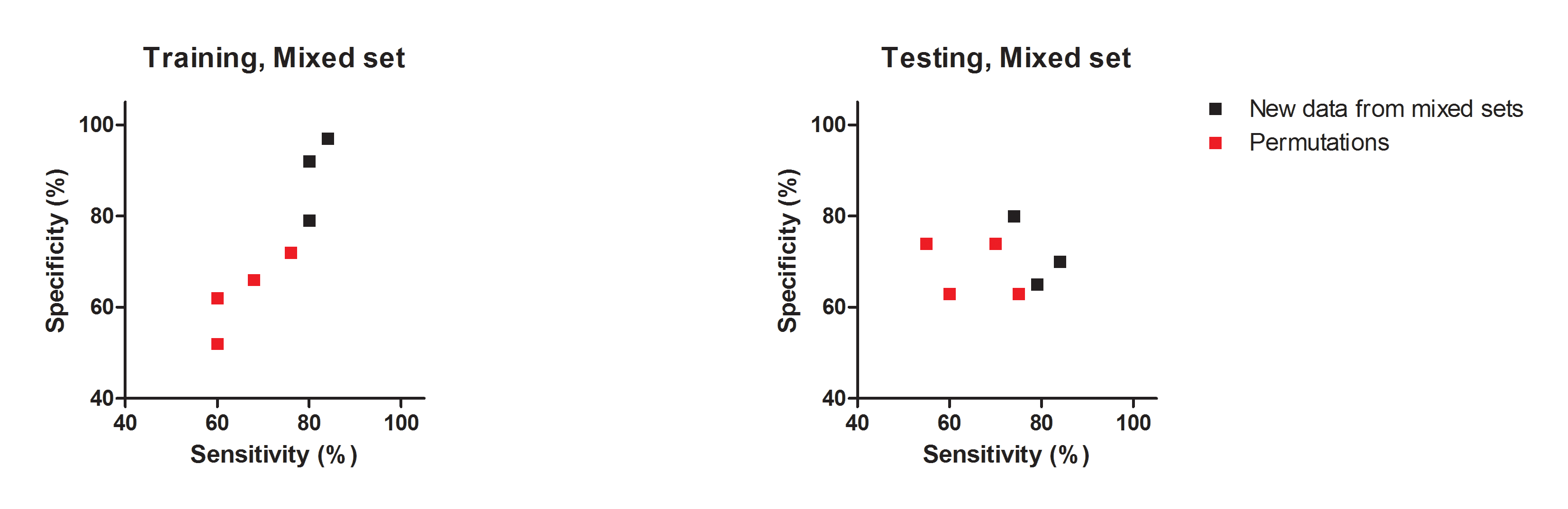

Supplement: Figure S4 — The performance of the prediction model was tested in Training and Testing sets, for both real data, and data in which the assignment of cases and controls had been randomized. This approach is the same as in Figure 6, except that each set was composed of samples drawn from a combination of both the Nelson-1 and Nelson-2 sets. We assessed three such combinations, and four permutations. (TIF) [file pone.0096029.s004.tif]
