# Supplementary material for: Peptides from the Variable Region of Specific Antibodies Are Shared among Lung Cancer Patients
Source: PLoS One. 2014 May 1;9(5):e96029. doi: 10.1371/journal.pone.0096029 (PMC4006902; doi:10.1371/journal.pone.0096029)
Supplement: Methods S1 — Additional detail on sample collection, the study population, IgG Fab Purification, NanoLC Orbitrap MS analyses and de novo sequencing. (DOCX) [file pone.0096029.s012.docx]

**Peptides from the variable region of specific antibodies are shared among lung cancer patients**

Dominique de Costa^1^, Ingrid Broodman^2^, Wim Calame^3^_,_ Martijn M. VanDuijn^4^, Christoph Stingl^4^, Lennard J.M. Dekker^4^, René M. Vernhout^5^, Harry J. de Koning^6^, Henk C. Hoogsteden^1^, Peter A.E. Sillevis Smitt^4^, Rob J. van Klaveren^1^, Theo M. Luider^4^

Departments of Pulmonology^1^, Clinical Chemistry^2^, Neurology^4^, Unit Trials & Statistics^5^ and Public Health^6^, Erasmus Medical Center, Rotterdam the Netherlands, StatistiCal BV, Wassenaar, the Netherlands^3^

**Supplementary Methods**

*Sample collection*

From 6,600 participants in the screen arm of the NELSON trial one serum tube have been collected during their first visit (baseline) at the screening center. All serum tubes have been centrifuged for 10 min at 1400 g and 4°C within 2 hours after collection and stored immediately in 3x 1 mL aliquots at -80°C according to a standard protocol for all screening sites. For this study we used a 1 mL serum aliquot of each of the 44 case and 49 control samples. The samples were thawed and subsequently aliquoted in 100 µL in 200 µL micronic tubes (Micronic B.V., Lelystad, Netherlands) and stored at -80°C. For this study we used one aliquot (100 µL).

*Study population*

Cases and controls had to have no history of cancer, the time interval between blood sampling and the diagnosis of lung cancer had to be <3 years and for the controls the follow-up duration after blood sampling had to be >2 years. The COPD status was either based on the GOLD lung function criteria (Pauwels et al. 2001) or if they answered to the questionnaire that they were known with chronic bronchitis or emphysema. Participants were classified as asbestos exposed if they answered to the questionnaire that they had performed once a job or activity with possible asbestos contact.

*IgG Fab Purification and NanoLC Orbitrap MS analyses*

Briefly, IgG was isolated from serum using the Melon Gel IgG purification kit (Pierce, Rockford, IL) and digested with immobilized papain (Pierce) into Fab and Fc. An anti-Fc affinity column was used to purify Fab from the digested mixture using the MicroLink Protein Coupling kit (Pierce) and further purified by SDS-PAGE.

Gel plugs were destained with 200 µL 100 mM NH_4_HCO_3_ in 35% (v/v) acetonitrile/15% H_2_O. Tryptic peptides were extracted from the gel by adding three times 50 µL 0.1% (v/v) TFA in 50% (v/v) acetonitrile/H_2_O. For nano-LC-MS/MS analysis, the dried peptides were dissolved in 20µL of a solution containing 98% ultrapure water, 0.1% (v/v) formic acid and 2% (v/v) acetonitrile. This mixture was stored at 4°C.

*NanoLC Orbitrap MS analyses*

4 µL of the digested Fab were loaded onto a C18 trap column (PepMap C18, 300µm ID × 5mm, 5 µm particle size, 100 Å pore size; Dionex) and desalted for 10 min at a flow rate of 20 µL/min 0.1% TFA (Biosolve, Valkenswaard, Netherlands). Next, the trap column was switched online to an analytic column (PepMap C18, 75µm ID × 150mm, 3 µm particle size and 100 Å pore size; Dionex). Peptides were eluted using a 180 min gradient with the following binary gradient: 0%-25% solvent A in 120 min and 25%-50% solvent B in the next 60 min, where solvent A consists of 2% acetonitrile and 0.1% formic acid in water and solvent B consists of 80% acetonitrile and 0.1% formic acid in water (all solvents used purchased from Biosolve). Column flow rate was set at 300 nL/min. The analytic column was then washed and equilibrated.

To identify the IgG Fab fragments we used a CAD fragmentation. High resolution full scan MS was obtained from the Orbitrap (resolution 30,000; AGC 1,000,000). MS/MS spectra were obtained by CAD fragmentation, and detection was conducted. MS/MS was performed on the top five masses in the full scan spectra. Dynamic exclusion was used, with a repeat count of one; exclusion duration was set at 3 min and exclusion width at +/- 5 ppm.

*De novo sequencing*

*De novo* sequencing was performed on the raw data files using software Peaks Studio 5.2 (Bioinformatics Solutions Inc.) For the *de novo* sequencing measurement the following settings were used: a parent ion tolerance of 10ppm and a fragment ion tolerance of 0.5 Da were allowed. Trypsin was set as enzyme. Methionine was used as a variable modification of oxidation (15.995) with a maximum of three peptides allowed.
